# Developing an Intelligent Monitoring Technology for Airport Stone Column Machines

**DOI:** 10.3390/s20113050

**Published:** 2020-05-28

**Authors:** Ke Tang, Haiwen Yuan, Jianxun Lv, Fengchen Chen

**Affiliations:** 1 School of Automation Science and Electrical Engineering, Beihang University, Beijing 100191, China; tangke@buaa.edu.cn (K.T.); yhw@buaa.edu.cn (H.Y.); 2 China Airport Construction Group Co., Ltd., Beijing 100621, China; 15811175020@163.com

**Keywords:** stone column machine, intelligent monitoring, GNSS, laser ranging sensor

## Abstract

Most of the construction machinery for vibro-sinking stone columns, which are widely used in China, needs to be improved in terms of degree of automation. Engineering quality control is mainly carried out post-inspection; consequently, it is difficult to control the construction quality in real time. According to the construction characteristics of traditional stone column machines, we established the theory and model for the real-time monitoring of stone column construction, as well as put forward an intelligent monitoring method for stone column machines. With the comprehensive application of critical technologies such as the Global Navigation Satellite System (GNSS) measurement technology, laser ranging sensors, and massive data processing, an intelligent data acquisition technique and associated monitoring equipment for stone column construction machines are developed. The data acquisition and storage of crucial construction parameters, such as pile depth, pile point co-ordinates, bearing layer current, and reverse insertion times, are realized. A large number of actual construction data are collected and the construction quality parameters of stone column machines are obtained. By comparison with third-party detection data, it is verified that the intelligent monitoring technique for stone column machines proposed in this paper is feasible.

## 1. Introduction

Stone columns are composite foundation reinforcing piles, with gravel (pebble) used as the primary material. They have the primary functions of replacement, compaction, and promoting drainage consolidation, and are mainly used in soft foundation strengthening, embankment slope strengthening, eliminating liquefaction in liquefiable sand soil, and eliminating collapsibility in collapsible loess. Construction of a stone column is a typical concealed project, its core construction mainly includes several steps, such as aiming point, vibrating pile, filling, vibration pulling, retaining vibration, reverse insertion, and finishing pile moving.

Stone columns were initially used for building up the arsenal workshop in 1835 in Bayonne, France. In 1937, the German company Keller invented the vibro-jet (vibroflotation) for sandy soil foundation compaction. During the early 1960s, the vibroflotation method was utilized in Germany to reinforce a clay soil foundation and form a stone column. Subsequently, the method has been widely applied in countries all over the world; as time has drifted by, a variety of different construction technologies have been developed, correspondingly.

Based on the diversity of construction methods, stone column composite foundations can be divided into the following types: Vibrating water jetting stone column composite foundations, dry-drilled vibration compaction stone column composite foundations, vibro-sinking stone column composite foundations, stone column composite foundations using the dynamic compaction replacement method, etc. These stone column composite foundations all lie within the range of loose material pile composite foundations; although their construction methods differ from the vibroflotation method, they still form compacted stone columns.

The construction machineries used in the vibro-sinking method to form the pile mainly include vibration machines, vibration hoppers, and vibration pile drivers composed of a vibrating lining tube. Vibro-sinking stone columns are formed under the vibration effect of the vibration driving hammer, where the lined tube is sunk down to the required design depth. After the lined tube is sunk into the soil layer, the soil layer surrounding it is compacted. Then, gravel is put into the lined tube and spread into the soil layer. Vibration and compaction are carried out in sections to form a larger-diameter stone column with further compaction of the soil layer between piles; after repeating this process many times, the stone column is completed.

At present, the most widely adopted method in a major part of China is the vibro-sinking stone column construction technology, in which the machinery and equipment applied is relatively traditional, belonging to the range of half-mechanization equipment. The majority of the construction machinery also needs to be disassembled before transportation to the project site and re-assembled (even requiring welding work) at the construction site. Labor monitoring of construction is required at the project site, which mainly has the following problems: (1) Control of the construction monitoring process is not comprehensive (i.e., increasing the frequency of random sample tests does not necessarily ensure the quality of every finished pile); (2) the labor costs are very high, such that 24-h of continuous monitoring cannot be ensured and there is always a period of under-qualified monitoring, which increases the possibility of low construction quality; (3) with traditional construction methods, there is a higher requirement for an enriched experience when assessing the index value during monitoring, where the experience itself cannot be specifically quantified, only relying on experienced construction personnel and inevitably increasing the labor costs; (4) the traditional methods of construction monitoring measurement are less precise, unable to obtain accurate statistics, and can only be achieved through the accumulation of workload, where the error increases with the accumulation, which has a huge impact on construction quality; (5) in the case of any unqualified pile positioning during construction monitoring, rectification of the work is inevitably required, thus holding up the whole process of construction and increasing the cost of construction; (6) the statistical data report lags behind the construction process, such that the data cannot be accounted for in real time and can only be sorted out by staff in a later phase. In this way, human factors can significantly interfere with the decision-making in the whole construction process.

In order to guarantee the construction quality and improve the management level, technical innovation is urgently needed to solve the construction monitoring problem for stone columns.

The field of site construction has always been a more traditional field. With the development of science and technology, the automatic control and monitoring of construction machinery have been realized by installing positioning and special sensors on construction machinery. At present, there are many automatic control products, such as intelligent grade control systems, intelligent compaction control systems, 3D piling control systems, 3D dynamic compaction control systems, groundworks machine control systems for drilling, dozer control systems, excavator control systems, paver control systems, etc. [1,2].

The Keller Company has developed instrumentation for stone column production, which can be instrumented with an on-board data acquisition system. Then, parameters and piling reports can be automatically obtained online [3].

In order to monitor the construction quality of airport stone column machines accurately and quickly, it is necessary to research and develop an intelligent stone column monitoring system with the characteristics of real-time use, continuity, automation, and high accuracy. It must automatically collect data and realize the real-time, dynamic, and nondestructive [4] measurement of the relevant parameters in the construction process, in order to better meet the actual needs of airport construction and management. It should realize an advancement of the stone column monitoring and control management mode from the traditional planar approach to a three-dimensional, comprehensive, and real-time information mode. With such technology, managers can obtain the dynamic construction information of airport construction stone columns quickly, comprehensively, and accurately. Thus, it can successfully control the construction quality and construction progress [5,6].

At present, the vibro-sinking stone column technology, which is widely used throughout China, is difficult to monitor in real time over the whole process. Based on this, first, the monitoring parameters and key parameters of vibro-sinking stone columns are analyzed in this paper, and then a monitoring hardware terminal and monitoring software are designed and developed. The proposed methodology is applied in the process of vibro-sinking stone column construction at an actual airport. The collected data is analyzed and compared with testing data from a third party to evaluate the ability of the proposed methodology in predicting the performance and quality of a stone column. The system launches measures to eliminate its impact, such that the production process is within a controlled state and the purpose of control and stable quality is achieved. Thus, the passive post-test is transformed into an active prevention process, which greatly reduces waste and improves project quality and efficiency. If there is no special description, any “stone column” mentioned later in this paper refers to a “vibro-sinking stone column”.

## 2. Analysis of Stone Column and Critical Sensor Technology

Christoulas et al. [7] carried out an experimental study on model stone columns, where the load–deformation response and failure modes of stone columns were evaluated directly. In addition, according to the model test results, a trilinear load–settlement relationship was proposed, which was directly used for design purposes. Castro [8] carried out numerical modeling of stone columns beneath a rigid footing. A set of systematic two- and three-dimensional finite element analysis methods were proposed to study the working performance of group stone columns beneath a rigid footing. A numerical analysis showed that the number of columns and their arrangement had little influence on the load–settlement curves. Xue et al. [9] analyzed the triaxial compressive behavior of geotextile-encased stone columns, where a series of large-scale triaxial tests on stone columns with or without geotextiles were carried out. The stress–strain relationship of encased stone columns was described as the superposition of a hyperbolic and linear model, which were used to simulate the behavior of an ordinary stone column under triaxial compression and uniaxial compression, respectively. Liu et al. [10] studied the rocking response of a shallow foundation strengthened by stone columns in a soft clay environment by numerical simulation. Stone columns are often used to improve the bearing characteristics of soft soil foundations [11]. Nav et al. [12] studied the mechanical behavior of ordinary stone columns and reinforced stone columns, as well as the effects of parameter detection.

Shao et al. [13] used a data acquisition and data recording system for vibro-sinking stone column construction to monitor, record, and display the vibrator penetration depth, energy consumption, gravel consumption, air pressure, and other parameters. The monitoring system continuously aids both the operator and the inspector. Based on TCP/IP data acquisition technology, wireless communication, a graphical user interface, and GPS satellite navigation and gyro technology, a vibro-sinking stone column monitoring system was developed to better monitor the construction quality of vibro-stone columns. In order to determine the properties of the soil at the site, it is necessary to sample the soil by drilling. The parameters of the basic engineering properties of the soil, especially the soil strength parameters, have been obtained [14].

At present, the influence of different parameters on the stress ratio of the working pile and the lateral displacement of foundation soil are typically studied by field tests and numerical analyses. Due to the characteristics of novel technologies and diversified construction methods, the non-standardization of quality testing methods and study of the working mechanism need to be combined with on-site tests, relying on strict monitoring of construction parameters to ensure the quality of the final product. There have been few studies on the digital and intelligent construction of stone columns, mainly pursuant to installing individual sensors to detect the construction parameters and control the quality.

The construction quality monitoring system for vibro-sinking stone columns designed and developed in this paper mainly considers such important parameters as the time of pile sinking, the time of pile pulling, pile point co-ordinates, pile sinking depth, the current of the stress-bearing layer, the number of times of reverse insertion, and the amount of stone filled. The measurement parameters are independent yet closely related to each other, and are described in detail in the following:

(1) Pile point co-ordinates

Error in the measurement of pile position or inclination of pile and improper pile body sinking technology can result in large deviations in the completed pile position, resulting in poor shear strength in the soil layer as well as locally over-intensive load. Thus, correct monitoring of the co-ordinates of each pile point is the basis for an appropriate online display of pile point information and counting of the number of pile points.

(2) Pile sinking depth

The pile depth of a stone column is mainly used in the general area around 10–20 m of depth in soft soil foundations. The pile length is determined according to the preliminary investigation data of design parameters, and construction units should carry out construction according to the actual geological conditions. When the pile bottom reaches relatively hard strata (i.e., stress-bearing strata), it is judged that the pile has reached the bearing layer, and the overall length of the pile at this time is the actual depth value of the sinking pile.

(3) Current of the stress-bearing layer

The current of the stress-bearing layer refers to the motor current value when the pile sinking pipe reaches the bearing layer. Due to the fact that the pile head is deep underground, there exists no accurate measurement method to confirm whether the pile pipe has reached the bearing layer. According to a long-term practice and experience, it is generally considered that the bearing layer can be basically determined as the depth at which the motor current increases obviously on a continuous basis, which can be considered as a control parameter for whether the depth of pile satisfies the design requirements.

(4) Other parameters

In the process of the construction pile pulling, in order to achieve a compaction effect on the pile body, the stone column machine should repeatedly accumulate, compact, and fill in with gravel; we refer to this as reverse insertion. Each time, the decline of the insertion depth operation should not be less than 30 cm, which can be deemed as valid reverse insertion. Pile continuity and compaction degree are evaluated as critical benchmark parameters for the quality judgment of reverse insertion; however, it is difficult to control in the process of actual construction. The statistical method is commonly hand-drawn, such that the artificial error is relatively high.

The filling of gravel refers to the total amount of gravel filled into the pile tube in the operation of pile sinking and pile pulling, which is mainly used as a metering control parameter in the actual construction.

The real-time monitoring system of construction quality for stone columns proposed in this paper is mainly aimed at evaluating quality control parameters through the measurement of construction quality parameters, in order to monitor the whole process of construction in an overall and three-dimensional manner, avoiding the risks associated with a single index error or failure and ensuring construction quality at a controllable level.

The intelligent monitoring technology for stone columns proposed in this paper mainly involves sensor technologies such as the Global Navigation Satellite System (GNSS), laser ranging sensor [15], weighing sensor, current transformer, etc.

GNSS [16,17] is a satellite navigation system, which can provide navigation positioning and timing services for users with high-dynamic and high-precision results under all weather conditions. It takes advantage of observations such as the pseudo-range, ephemeris, and launch time of a group of satellites and requires knowledge of the user clock error. The current system includes GPS in the United States, GLONASS in Russia, Galileo in the European Union, and Beidou in China. GPS (Global Positioning System) is an all-weather navigation and positioning system, which provides positioning and timing services. While multiple systems provide independent navigation and positioning services, users can make full use of multisystem resources to achieve better (or, at least, comparable) services than single systems [18].

A high-precision GPS measurement must use carrier phase observations. The RTK (real-time kinematic) [19,20] positioning technology is based on carrier phase observations of real-time kinematic positioning technology, which can provide real-time three-dimensional positioning results in a specified co-ordinate system, achieving up to centimeter accuracy. Therefore, this paper uses the RTK carrier phase real-time difference technology to provide a centimeter-level, high-precision positioning for stone columns monitoring.

At present, laser ranging sensors also play an important role in the field of civil engineering [21,22]. A laser ranging sensor first emits a laser pulse from its laser diode towards the target. After reflection from the target, the laser is scattered in all directions. Part of the scattered light is returned to the sensor receiver, which is received by the optical system and imaged on an avalanche photodiode. An avalanche photodiode is an optical sensor with an internal amplification function, such that it can detect feeble optical signals. The target distance can be measured by recording and processing the time between the light pulse and the return of the received signal. Since Hughes Aircraft Corporation developed the world’s first laser rangefinder in the 1960s, laser rangefinders have been widely used in various industries. It provides significant advantages for all kinds of non-contact measurements.

A weighing sensor is a device which converts a mass signal into a measurable electrical signal output. In all kinds of weighing and mass-measuring systems, the integrated error is usually used to derive the integrated control sensor accuracy. It is convenient to select a weighing sensor corresponding to a weighing device with specified precision. The weighing transmitter, also known as the weight transmitter, is a transmitter which converts a physical quantity into an electrical signal and converts the output of a millivolt signal into a standard Direct Current (DC) signal using isolation amplification [23].

A current transformer is an instrument which converts a large current on the primary side into a small current on the secondary side, based on the principle of electromagnetic induction. Current transformers are comprised of a closed core and winding. On its primary side, the number of winding turns is few, and the string is in the circuit where the current needs to be measured. A Roche coil can be used as an ideal current sensing element [24]. 

The main control box terminal unit of the proposed system includes modules such as a GNSS receiver, an antenna, a power converter, a water- and shock-proof stainless steel tank, an alternating current sensor, weighing sensors, infrared laser ranging sensors, an industrial flat-panel display device, etc. Each sensor, either connected by a cable or wirelessly, can communicate with the data processing module, where the wireless methods include 4G, Bluetooth, and ZIGBEE. The main control box computer contains a 4G phone card and, at the same time, can transmit data through the wireless network mode to the monitoring platform. The stone column intelligent monitoring system developed by this paper involves the main sensor measuring principles and technical indices of monitoring parameters shown in Table 1.

## 3. Design and Development of Intelligent Monitoring Equipment

**Figure 1:**
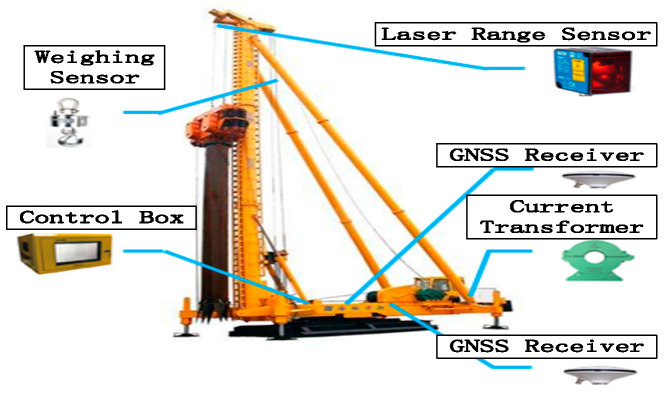
Hardware device composition.

In this paper, the intelligent monitoring of stone column construction machines is studied. Firstly, the monitoring parameters are analyzed and suitable sensors are selected. A set of model algorithms for the real-time collection of important parameter information of the stone column machine are designed and developed by using GNSS RTK positioning, laser sensing, and internet of things transmission technologies. The quality monitoring system is used to collect the related parameters in terms of a specific algorithm. After the intelligent monitoring system was designed and developed, an experiment was carried out at a stone column construction site. The hardware device composition for stone column construction monitoring is shown in Figure 1.

**Figure 2:**
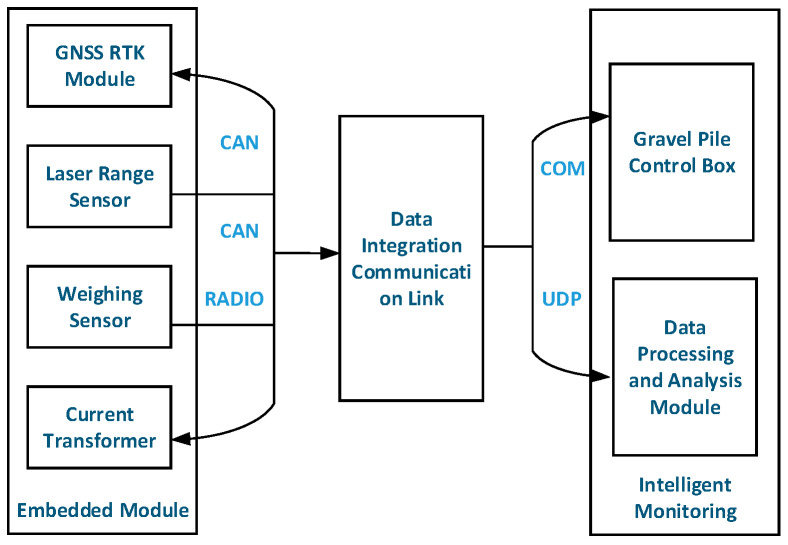
Stone column monitoring frame.

The stone column monitoring system consists of an acquisition sensor, a GPS module, a transmission system, a power supply, a back-end management system, etc. The GNSS positioning antenna is installed on the foundation treatment equipment of the stone column machine to realize the management of construction position and construction time. Using the current transformer, we can realize the measurement of the electrical current value of the equipment. We use the laser sensor equipment to achieve the monitoring of the depth of the foundation treatment and the reverse insertion number. The weight gauge (tension sensor) can be set up to monitor the feed quantity. The structure of the stone column monitoring equipment is shown in Figure 2.

CAN (Controller Area Network) is a serial communication protocol used for real-time application buses. It can use a twisted-cord cable to transmit signals, which is one of the world’s most widely used field buses. The robustness of the protocol facilitates its extended application to other automation and industrial functions. RADIO (RADIO frequency unit), which is responsible for the sending and receiving of data and voice, is characterized by short distance and low power consumption. Bluetooth antennas are generally small in size and light in weight and belong to the class of microstrip antennae. The serial communication port is abbreviated as COM (cluster communication port). Data transfer between computers or a computer serial port and a terminal can use two schemes: Serial communication and parallel communication. As the serial communication mode has the characteristics of less line and low cost (especially in remote transmission, to avoid the inconsistency of multiple lines), it has been widely applied. UDP (User Datagram Protocol) is an Internet Protocol which supports a connectionless transmission method, such that it is not necessary to establish a connection when sending an encapsulated IP data package.

Based on the above conditions, in the process of product development, the complex and harsh construction conditions of the stone column machine were fully taken into account to improve the physical index requirements of various sensors, such as the necessity for it to be waterproof, dustproof, and shockproof. At the same time, data fusion, wave filter, and analysis algorithms were added to the software itself, in order to eliminate abnormal values and accurately reflect the normal working information of the equipment.

## 4. Design and Development of the Intelligent Monitoring Software

The intelligent monitoring software for the stone column machine can receive data collected by the front-end monitoring hardware equipment in real time, providing working information of the monitoring equipment. It can also provide a construction information report.

### 4.1. Monitoring Software Framework

Under the unified and general Geographic Information System (GIS) platform, the software adopts a multilayer architecture organization and is compatible with various large database systems, following the principle of openness and standardization. By adopting the object-oriented data model and componentization GIS software technology, the system is designed with suitable open-function interfaces and data interfaces, ensuring that the system has good expandability and ductility. Due to the design idea of high cohesion and low coupling (cohesion refers to the degree of correlation between parts within a module and coupling refers to the degree of correlation before each module; high cohesion and low coupling is a concept in software engineering), the mixed development structure of B/S + C/S (Browser/Server and Client/Server) was adopted to realize the unified management and monitoring of each functional module of the stone column. It realizes remote real-time visual monitoring and provides various query and analysis functions. Through the analysis of various data, the weak points of the project can be found in real time, which can provide a basis for critical inspection. It can improve the overall efficiency and level of airport construction, management, and operations.

### 4.2. Monitoring Software Functions

As the core hub of the system processing, the monitoring software mainly includes the unified and integrated management of data collected from the front-end stone column machine and tools. The back-end monitoring software mainly completes data preservation, analysis, and backtracking. The results are presented to the manager by graphical means, such as a report form and a statistical chart, which provides a basis for the manager to control the construction status macroscopically.

The monitoring software is based on the cloud GIS technology architecture and applied in the construction stage of airport construction. It aims to realize the visualization of the monitoring process of stone column construction and real-time quality monitoring. Using the proposed construction monitoring software technology, manual management error in the construction at this stage can be avoided. Construction information management can be effectively realized, while the management level can be improved and the management cost reduced.

#### 4.2.1. Parameter Monitoring

The monitoring parameters include pile sinking depth, bearing layer current, filling amount, reverse inserting times, and state monitoring. The map displays the piling position information of the current working layer and uses different colors to represent the parameter information of each piling position.

#### 4.2.2. Stone Column Data Monitoring

By obtaining the co-ordinate information of the construction section, a GIS geographic layer is established and meshed such that each grid is small enough to mark the grid position with the geodetic co-ordinates of the grid center point. In the process of real-time monitoring, the construction range of construction pile points can be determined from each new set of effective pile construction data obtained. The co-ordinates of the pile pipe can be combined with the size of its diameter, in which the pile pipe can be accurately drawn on the GIS map. At the same time, the construction parameters under the co-ordinate point, such as the depth of the sinking pile, the current of the load layer, and the number of reverse insertions, can be assigned; such that a point or area can be inspected and analyzed in the map.

The map marks different working conditions with differently colored icons. When clicking the icon, an animated simulation of the work and the real-time working data of the stone column are displayed, where a stoppage animation simulation is presented in the case of work stoppage.

**Figure 3:**
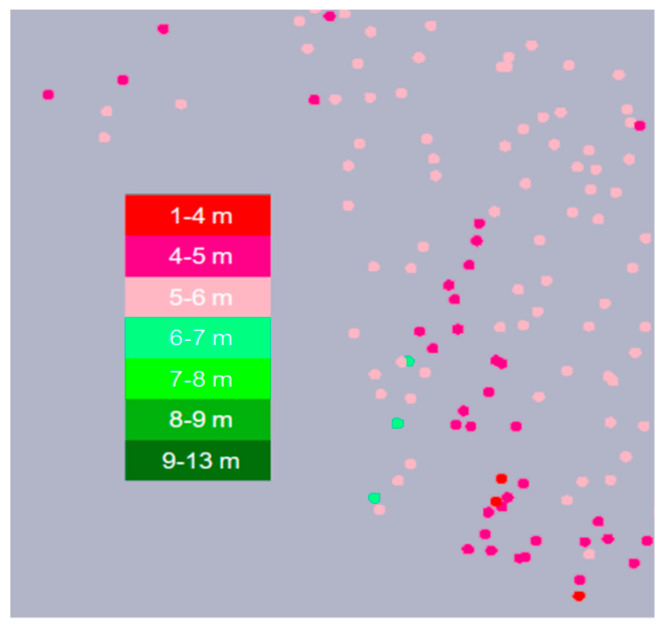
Pile depth monitoring.

For pile depth monitoring, we use different colors to display different pile depths in real time, where the unit is a meter. According to different depth ranges, all monitoring stone columns are given corresponding colors, which are indicated by the following color classification: The depth of 1–4 m is marked with red, 5–6 m with pink, and 9–13 m with green, such that the construction situation is clear and intuitive. Project managers can remotely grasp and scientifically adjust the project construction schedule, as well as rectify the weak areas detected in the system duly, in order to ensure the project quality. The monitoring effect of pile depth is shown in Figure 3.

**Figure 4:**
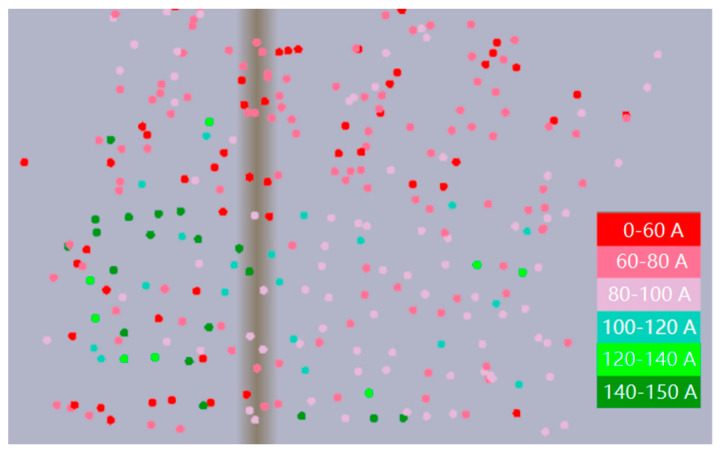
Bearing layer current monitoring.

In the bearing layer current monitoring, all monitored stone columns are given corresponding colors according to the current range of different load layers and marked by color. For example, a red solid circle is used for pile points in the 0–60 A range, a pink solid circle is used for pile points in the 80–100 A range, and a green solid circle is used for pile points in the 140–150 A range, in order to convey the current distribution of all construction stone columns in real time. An example is shown in Figure 4.

**Figure 5:**
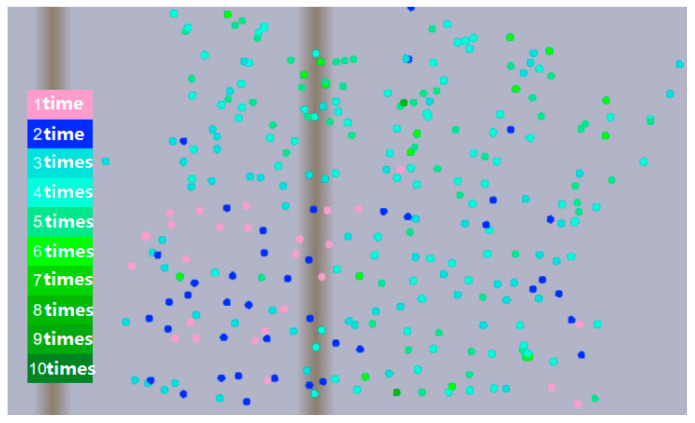
Reverse insertion monitoring.

In reverse insertion time monitoring, all monitored stone columns are demonstrated with corresponding colors, according to different times of reverse insertion, which are indicated by color; for example, one reverse insertion is indicated with a pink solid circle, and 6–10 reverse insertions are indicated by green solid circles. In this way, we can obtain a real-time understanding of the current reverse insertion times of all construction stone columns. An example is shown in Figure 5.

#### 4.2.3. Stone Column Information Query

A variety of data query methods, such as point selection, box selection, condition selection, and other data queries, can be used to query stone column monitoring information. The query results can then be exported using Excel.

#### 4.2.4. Stone Column Information Statistics

**Figure 6:**
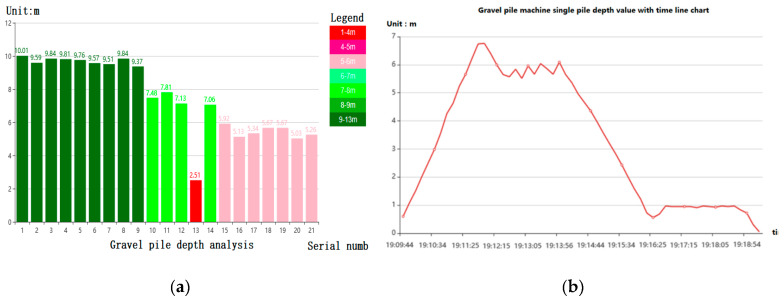
Selection and statistics of stone column monitoring: (**a**) Statistics of driving depth of a stone column; and (**b**) broken line chart of a single pile depth with the time of stone column machine.

The software can realize point selection, box selection, or statistics of monitoring information of the stone column machine in accordance with the period, number of stone column machines, and other conditions. Queries or statistics according to different conditions can be used to obtain the corresponding attribute information; as shown in Figure 6a, where we selected 21 pile points on the GIS map, queried the pile depth of each pile, and drew a column chart. From the legend, it can be seen that, for different pile depth ranges, the color is different. When selecting a pile point on the GIS map, relevant parameter information of the pile point can be queried, such as multiple pile points selection; as shown in Figure 6b, where the variation of depth of a single stone column was queried over time. Similarly, other monitoring parameters can also be queried and statistically analyzed. All queries, statistical property sheets, and statistical graphs can be exported to Excel.

Figure 6b is a line chart showing the variation of depth of a single pile over time. It can be seen that, at the beginning of piling, the pile depth increases with time. When the bearing layer is hit, it cannot be driven down any further, and the depth of the pile reaches its maximum. Then, pulling of the pile and reverse insertion of the pile begun, and the pile depth became smaller. When the pile head was pulled out of the ground, the pile was completed and the pile depth was restored to zero. Then, we can move to the next pile point and start piling.

#### 4.2.5. Analysis Report Output

Parameters can be printed and exported in Word and Excel, based on a selected area or specified time or machine. Construction reports based on custom templates can be exported.

## 5. Data Processing and Analysis

### 5.1. Test Case

The system developed in this paper was verified in the actual construction environment of Chengdu Tianfu international airport. The pile test design of the stone column was an equilateral triangle pile with a diameter of 600 mm, a distance of 1.5 m at the pile center, and a design value of 7 m at the pile depth. Chengdu Tianfu international airport is currently one of the most complex, in terms of airport ground treatment, where the site field is mainly of shallow gully landform, the terrain is east–west high–low, meteorological north generally low, relative elevation difference within 50 m, and landfill and excavation earthwork to date totals 180 million cubic meters. The whole area is 20.2 square kilometers, with more than 370 hills, hundreds of gullies, and the foundation treatment is extremely difficult. Tianfu airport has been divided into 11 bid sections. Taking one bid section as an example, the monthly peak pile intensity of stone column machines in the bid section was estimated to reach more than 20,000 in quantity, with an average depth of more than 7 m. If, during the peak period of foundation treatment construction, the stone column machines in all fields are operated at the same time, quality control will be very difficult.

**Figure 7:**
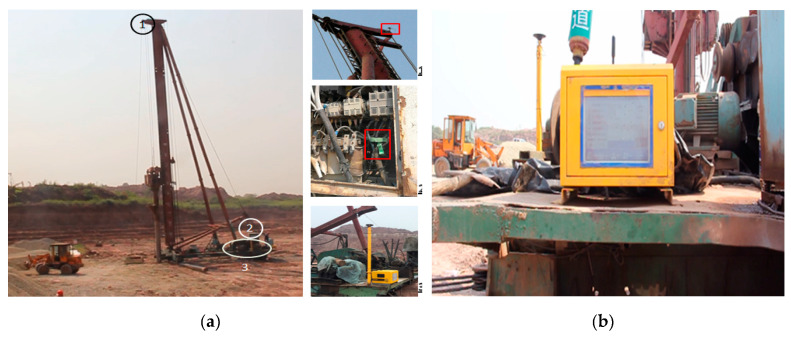
Construction site of the stone column monitoring: (**a**) Location of the monitoring technologies on the stone column machine; and (**b**) real-time display of the monitoring parameters.

The construction site piling was as shown in Figure 7a, where installation position 1 is the laser sensor, position 2 is the current transformer, and position 3 is the Beidou positioning double antenna and main control box. The wiring of the machine body does not affect the normal operations of the vehicle, and fixing of the module components avoids destructive operations on the vehicle, such as drilling and welding of the machine body. The display of construction technical parameters of pile driving obtained on-site are shown in Figure 7b, and some of the data intercepted for comparative analysis with the on-site construction records are listed in Table 2.

Based on the analysis of the data measurement comparison results in Table 2, three conclusions can be drawn regarding the site construction situation: (1) After verification with the site intelligent RTK+ survey manual, the accuracy of the pile point co-ordinate positioning and measurement was at a centimeter level; (2) the error of measuring pile sinking depth by laser sensor was less than 0.1 m, which meets the construction technical requirements; and (3) the construction quality of the digitized construction quality monitoring system installed on the stone column construction machine was better than that of un-furnished machines in the same working area. Furthermore, the number of reverse insertion times and the current value of the load-bearing layer basically met the design requirements.

Based on the analysis of the data measurement comparison results in Table 2, three conclusions can be drawn regarding the site construction situation: (1) After verification with the site intelligent RTK+ survey manual, the accuracy of the pile point co-ordinate positioning and measurement was at a centimeter level; (2) the error of measuring pile sinking depth by laser sensor was less than 0.1 m, which meets the construction technical requirements; and (3) the construction quality of the digitized construction quality monitoring system installed on the stone column construction machine was better than that of un-furnished machines in the same working area. Furthermore, the number of reverse insertion times and the current value of the load-bearing layer basically met the design requirements.

### 5.2. Analysis of Sample Data of the Intelligent Monitoring Pile Sinking Process

#### 5.2.1. Pile Sinking Depth

**Figure 8:**
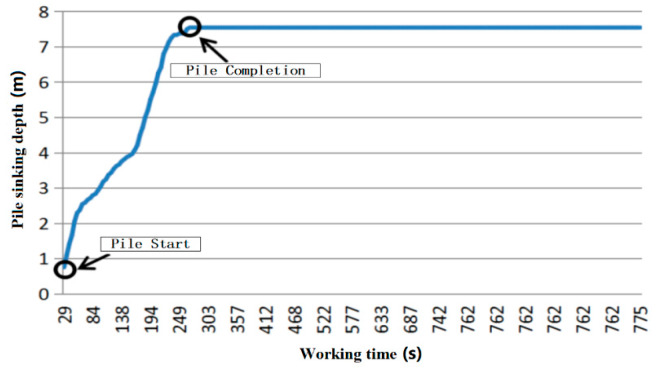
Depth of pile changes with the time curve diagram.

In Figure 8, the working time and pile sinking depth at the beginning and completion of the pile-sinking are shown. The construction started at 29 s and the final pile sinking depth of 7.54 m was reached at 263 s. Therefore, the whole pile sinking process lasted 4 min.

#### 5.2.2. The Current of the Bearing Layer

**Figure 9:**
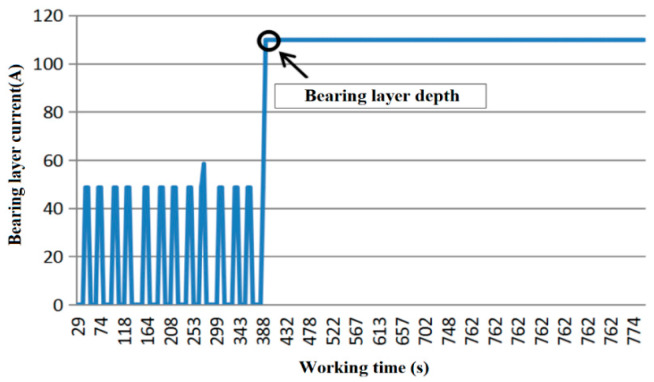
The current curve of the bearing layer varies with time.

Another index which is used to judge whether the pile is completed is whether the current of the bearing layer reaches a stable value. During the actual construction, due to the vibration of the stone column and field disturbances, the bearing layer current generated by the current sensor during the construction (0–394 s) fluctuates. It fails to reach a stable value, indicating that the sinking pile has not been completed. Finally, at 394 s, the current of the load-bearing layer reaches a stable value (109.83 A) which does not change with time, indicating that the pile-sinking process had completed, as shown in Figure 9.

### 5.3. Analysis of Sample Data of the Intelligent Monitoring Pile Pulling Process

The whole process of pile pulling is analyzed below, including the pile pulling depth, the reverse insertion times, the reverse insertion length, the reverse insertion depth, and the reverse insertion current.

#### 5.3.1. Pile Pulling Depth

**Figure 10:**
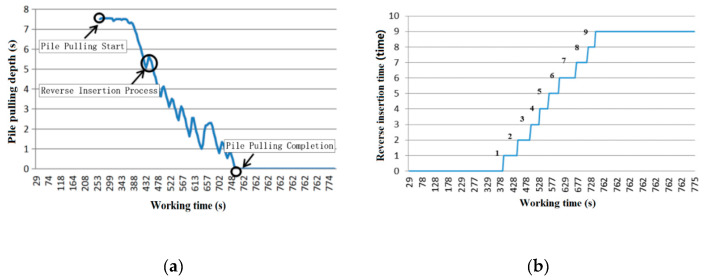
Change of pipe pulling depth and reverse insertion times with time: (**a**) Change curve of pile depth with time; and (**b**) number of reverse insertions.

The pulling speed should be uniform and controlled within 1 m/min, the depth of reverse insertion per 1 m of tube should not be less than 30 cm at 2–6 m from the surface, and the depth of reverse insertion should be no less than 50 cm. As can be seen from Figure 10a, after the pile was completed, the pile was pulled up at a uniform speed (the slope is the speed of pulling the pile), after some time at the original depth, until the construction process was completed. The curve reflexes represent a back-stepping process, as shown in Figure 10b. The change condition of reverse insertion times (as time changes) can be observed.

#### 5.3.2. Reverse Duration

**Figure 11:**
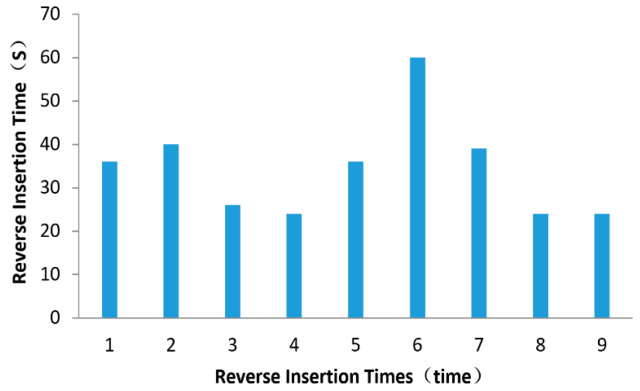
The reverse duration of the stone column.

In Figure 11, the reverse insertion times and the use time are shown. The construction of this point had a total of nine reversal times, with a total duration of 309 s and an average reverse insertion time of 35 s, which met the requirements within the average extubation speed.

#### 5.3.3. Reverse Depth

**Figure 12:**
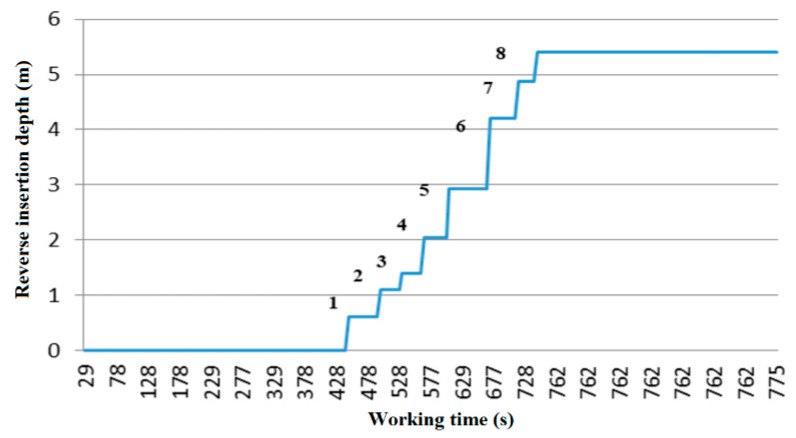
Inversion depth varying with time.

As shown in Figure 12, the depth and time of each inversion were calculated using cumulative inversion. The vertical height of the curve is the inversion height. The first three inversion depths are more than 6 m from the ground, and the contrast depth is no less than 30 cm. Then, the fourth inversion depth is less than 6 m from the ground, and the inversion depth is no less than 50 cm.

#### 5.3.4. Reverse Current

**Figure 13:**
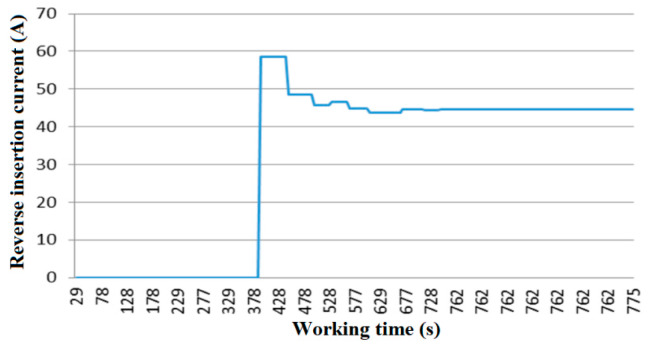
Change curve of reverse plug current.

As shown in Figure 13, the reverse insertion current is maximal in the first reverse insertion at 378 s. Then, the reverse current decreases gradually with the depth of pile pulling and finally reaches a stable minimum, indicating the completion of pile pulling.

### 5.4. Sample Data Analysis of Stone Column Construction Process

**Figure 14:**
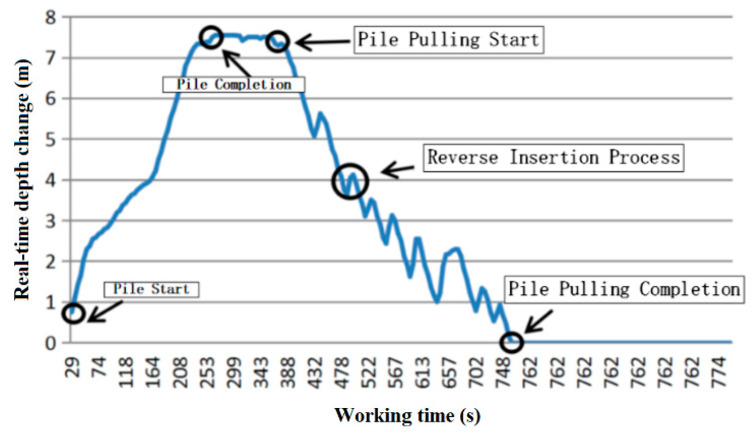
The whole process curve of stone column formation.

As shown in Figure 14, when the load bearing layer is reached, the pile is pulled out and reverse insertion is carried out according to the regulations. As the pile is pulled out, the depth of the pile becomes smaller and smaller until it is completely pulled out. By analyzing the digital monitoring data and constructing the whole process diagram of stone column construction, the quality of the construction process can be observed. Therefore, the construction process can be effectively managed and the traceability of the construction process can be improved.

It can be seen, from the variation of pile forming depth, that the pile forming processes of most stone columns in different regions met the design requirements. In the actual construction process, there are some non-standard construction operations for stone columns, such as fast pile forming and waiting for a period of time after the completion of pile sinking. Through the monitoring system, such non-standard piles can be visually screened and taken into consideration when testing.

### 5.5. Heavy-Duty Dynamic Sounding Detection and Analysis

#### 5.5.1. Heavy-Duty Dynamic Sounding Principle

The basic principle of the cone dynamic penetration test can be explained mathematically, in accordance with the conservation theorem of energy [25].

Kinetic energy: When the hammer is struck, the wear heart hammer is raised to a certain height by hand and then let go, such that it falls freely. The hammering energy produced by the piercing heart hammer before contact with the anvil hammer pad can be expressed in the form of kinetic energy:

(1) *E_m_* = (1/2)*Mv*^2^,

where *M* is the quality of the core hammer and *v* is the speed at which the drop hammer falls freely before hitting the feeler rod.

Hammer energy can also be expressed as the conversion from gravitational potential energy:

(2) *E_m_* = *MgH* × *η*,

where *H* is the hammer drop distance, *g* is the acceleration due to gravity acceleration (taken as 9.8 m/s^2^), and *η* is the drop hammer efficiency (this is when adopted with an automatic friction release device, such as a rope or reel).

Hammer energy: Considering a series of factors, such as drop hammer mode, hammer eccentricity, head material, size, shape, guide rod friction, rod transmission energy efficiency, etc., the ideal hammer energy *E_m_* can be transformed in many ways. The specific flow relationship is as shown in Equation (3):

(3) *E_m_* = *E_e_* + *E_p_* + *E_c_* + *E_k_* + *E_f_*,

where *E_e_* is the energy consumed by elastic deformation of soil, *E_p_* is the energy consumed by plastic deformation of soil, *E_c_* is the energy lost by deformation of the probe, *E_k_* is the energy lost in collisions between the percussion hammer and a feeler, and *E_f_* is the energy consumed in overcoming rod-side frictional resistance.

The energy used for soil deformation *E_e_ + E_p_* (i.e., the energy used to overcome the resistance of the soil) is called the useful hammer energy, which only accounts for part of the total bond energy—usually about 60% of the ideal bond energy *E_m_*. 

When a certain penetration depth is specified, a heavy hammer with particular specifications (i.e., prescribed probe section, cone angle, and mass) and a specified falling distance should be used. At this time, the number of hammers of dynamic penetration directly reflects the dynamic penetration resistance of the soil. This can directly reflect the compaction degree and mechanical properties of the soil. Therefore, the hammer number of penetration depth is often used as an evaluation index for the dynamic penetration test in engineering.

#### 5.5.2. Correction Analysis of the Length of Dynamic Touch Probe Rod

When a dynamic contact test is used to determine the bearing capacity of a dynamic compaction foundation, the most influential factor is the length of the probe rod. The main influence of the length of the feeler rod is essentially the transmission and dissipation of the energy of the dynamic feeler hammer. Therefore, in the application of dynamic penetration test results, the number of hammer-strokes must be modified for rod length. Taking heavy cone dynamic sounding as an example, the number of measured hammers can be corrected by the following formula [26]:

(4) *N*_63.5_ = *αN′*_63.5_

where *N′*_63.5_ is the correction of the number of front hammering, *N*_63.5_ is the corrected hammering number, and *α* is the probe rod correction factor.

Correction coefficients of rod length under different rod lengths and hammer numbers are shown in Table 3.

**Figure 15:**
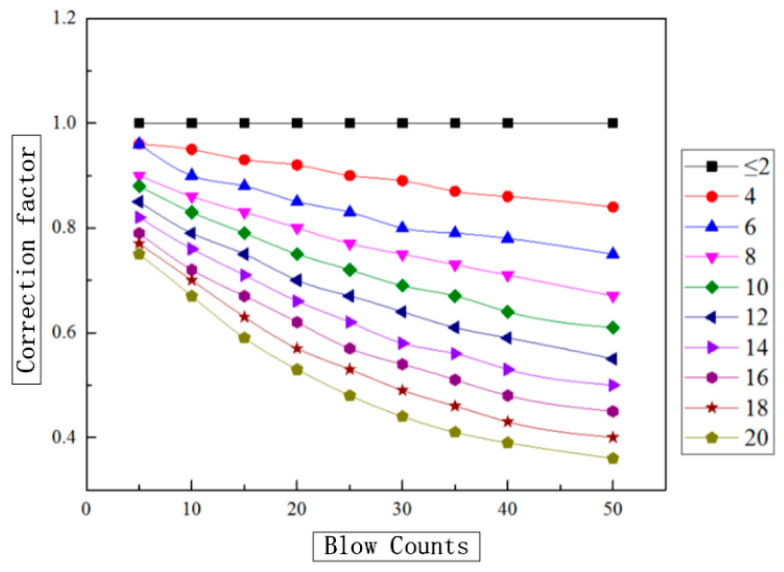
The bar length correction coefficient varies with the number of hammers.

Based on the data of Table 3, the relationship graph between the correction coefficient and the measured hammer number can be plotted. With a specified rod length, this relationship (correction coefficient and the rod length) is shown in Figure 15.

As can be seen from Table 3 and Figure 15, the minimum correction coefficient is 0.36, indicating that the influence of rod length can reach a severe degree, which should not be ignored in engineering applications. When the hammering value *N′*_63.5_ is the same, for different probe lengths, the smaller the rod length is, the bigger the correction coefficient is and the more realistic the test value is. When the rod length is less than 2 m, the correction coefficient is equal to 1; that is, there is no need to modify it.

With an increase of the hammering value *N′*_63.5_, the correction coefficient decreases. With the boundary of *N′*_63.5_ = 25, when the slope of the correction coefficient curve *N′*_63.5_ < 25 is more significant, the correction coefficient decreases rapidly with an increase of the hammering number. When the slope of the curve *N′*_63.5_ > 25 is gentle and the correction coefficient decreases with an increase of the value *N′*_63.5_, this phenomenon is more apparent when the length of the rod is more significant.

The boundary of the soil layer can be divided by dynamic contact data [27]. When the hammer number changes from small to large (i.e., soft soil to hard soil), the depth of the layer can be 2–3 times the diameter of the probe under the minimum of the last hammer number of the soft soil layer. When the hammer number is reduced from large to small (i.e., hard soil layer into soft soil layer), the stratification boundary is 2–3 times the probe diameter above the minimum point of the first hammer number in the soft soil layer [28].

The average value and coefficient of variation of the penetration index of each soil layer are calculated by single-hole statistics; then, the results are calculated by the thickness weighted average method. In statistics, individual outliers should be excluded from the “look-ahead” and “lag time” broad test points [26]. The final results were as follows: The revised minimum value was four strokes. The average value was 13.4 strokes, and the average value of 1–6 m ranges below the reinforcement surface (without cushion) was 7.1 strokes, which met the requirements.

To evaluate the compaction degree of foundation soil based on the data of the third inspection results, the third inspection results are shown in Table 4.

**Figure 16:**
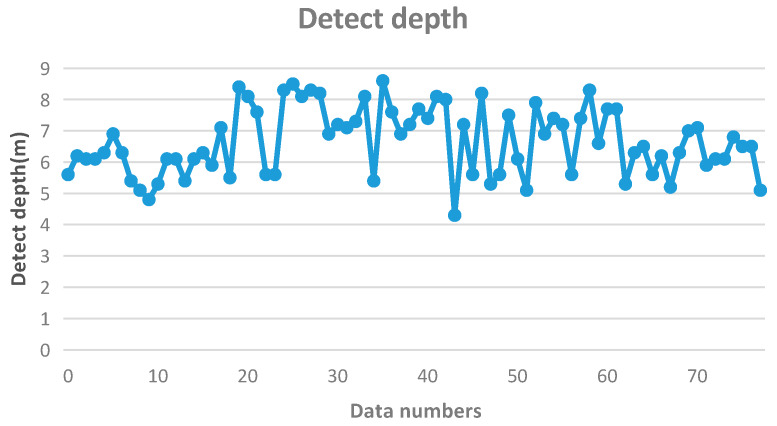
Detection depth of heavy power touch.

According to the test results, the compaction degree of foundation soil should be evaluated as follows: The revised minimum value should be no less than three strokes, the average value should be no less than six strokes, and the average value of the 6 m range below the reinforced surface (without cushion) should be no less than five strokes. The statistical test data are shown in Figure 16.

It can be seen, from Figure 16, that the detection depth of heavy power touch ranged from 4 to 9 m; that is, the rod length was between 4 and 9 m. Therefore, the actual results need to be corrected, according to the rod length and the hammering number.

**Figure 17:**
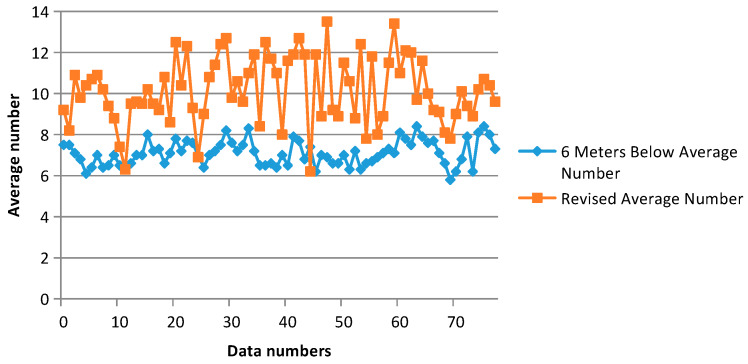
Fixed average pre-and post-strike graph.

It can be seen, from Figure 17, that the average number of strokes before revision was in the range of 6–8. Due to the difference in rod length and hammering number, the corrected average number was in the range of 8–13. The design requirements were as follows: The revised minimum should be no less than three strokes, the average value should be no less than six strokes, and the reinforcement surface (without the cushion layer) below a 6 m range average (beyond ten hits should be eliminated) should be no less than five strokes; it can be observed that the above results met the design requirements.

### 5.6. Analysis Summary

Utilizing the proposed digital monitoring system, the critical parameters of a stone column construction machine can be monitored. The weak parameters in the construction process of a stone column can be determined, and the construction quality can be ensured through the detection of the heavy dynamic touch. Through the analysis of the construction parameters, combined with detection methods such as heavy dynamic contact detection, the construction quality can be guaranteed.

## 6. Conclusions

In order to improve the quality and accuracy of monitoring in the construction of stone columns, we developed an intelligent monitoring technology for stone column construction in the airport context, including sensor selection, data collection, and integration of each sensor at problematic points. In this paper, we have detailed the theoretical research on and analysis of the construction principles of stone columns, analyzed the parameters to be collected by the intelligent monitoring system for stone column machines, and studied and developed the related intelligent monitoring equipment. A GNSS receiver, a laser ranging sensor, a weighing sensor, and a current transformer were installed in the relevant parts of the stone column construction machine to realize the automatic collection of the critical parameters. Finally, through data processing and analysis of the collected construction parameters, the correlation between monitoring parameters and requirements can be established and the weak parameters in the construction process can be determined. Thus, construction quality can be ensured by combination with the heavy dynamic contact test. It was proven that the proposed intelligent monitoring system for stone column machines is feasible, making the monitoring of the stone column machine digital and automatic. This makes the construction data precise and the construction process transparent, providing robust data support and ensures high-quality management and operations in future airport engineering. The field test results demonstrate that the system hardware has good seismic resistance, good data integrity, real-time performance, and high reliability. The accuracy of pile location monitoring reached up to a centimeter level, and the measuring error of pile depth was less than 0.1 m. This technology can fully reflect the construction process of a stone column machine. In this paper, we studied stone column construction quality by use of a real-time monitoring system which is not affected by weather conditions, can operate around the clock in foundation treatment engineering situations, and transforms the historical long-term reliance on hand-collected data for the construction management of stone column machines into an advanced and automated real-time process. Furthermore, the system realizes that data storage, data processing, and feedback analysis in a virtuous cycle, has a broad application prospect, and promotes moving the traditional industry technologies and management levels in an important development direction. The proposed system can provide powerful data support and guarantee the efficient management and operations of future airport engineering projects.

## Figures and Tables

**Table 1:** Technical parameters of sensors.

Sensor Type	Measurement Principle	Technical Index	Monitoring Parameters
GNSS receiver	Realizes the disintegration of real-time dynamic wave carrier phase difference to calculate the positioning co-ordinates and calculates the co-ordinates of the current pile driving points by using the dual antennas.	GPS: Synchronous L1 C/A L2E L2C L5 GLONASS: Synchronous L1 C/A L1 P L2C/A and L2 P/A BDS (beds): Synchronous B1 B2 B3 SBAS: Synchronous L1 C/A L5 real-time dynamic (RTK) horizontal: 10 mm + 1 ppm RMS; vertical: 20 mm + 1 ppm; RMS seismic value: 10–400 Hz 5g	Pile point co-ordinates Pile start/finish time Start/end time of pile pulling
Laser range sensor	Measuring sensor receives echo installed at the top of a pile pipe reflector and the distance between the sensors, in order to calculate the depth of the pile and analyze the inverse calculation of depth change. The Sick company’s DT50 series laser sensor was selected.	Resolution: 0.1 mm Measuring accuracy: ±10 mm Output Frequency: 50/25/10 Hz Service life: 100,000 h Operating voltage: 12–30 V	Pile depth Times of Insertion
Solenoid CT	The large current in the measurement circuit can be converted into a standard small current that can be measured by using the ratio relation of current transformer.	Current range: 500 A Measurement accuracy: 1 A Power consumption: <0.5 w	Pile driving current Piles pulling current Bearing Layer current
Wireless weigh sensor	The pin sensor is installed on the pulley and the steel rope pulls up the hopper. The weight of the stone can be measured by the pressure of the steel rope.	Weighing range: 5000 kg Indexing value: 5 kg Wireless transceiver distance: About 200 m	Filled gravel quantity

**Table 2:** Comparison of construction data and field construction record.

Co-Ordinate A	Co-Ordinate B	Pile Depth			Bearing Layer Current			Reverse Insertion Times		
		Monitoring Record	Site Record	Deviation	Monitoring Record	Site Record	Deviation	Monitoring Record	Site Record	Deviation
**3492.532**	7400.268	7.14	7.2	−0.06	239.72	235	4.72	3	3	0
3494.326	7400.251	7.19	7.2	−0.01	179.66	180	−0.34	4	4	0
3496.07	7400.232	7.18	7.1	0.08	227.37	230	−2.63	4	4	0
3497.956	7400.273	7.37	7.4	−0.03	204.28	200	4.28	5	4	1
3499.618	7400.261	7.48	7.5	−0.02	181.81	185	−3.19	3	3	0
3501.118	7400.287	7.78	7.8	−0.02	189.59	190	−0.41	5	5	0
3502.893	7400.284	7.89	7.9	−0.01	228.53	230	−1.47	3	4	−1
3504.658	7400.307	8.12	8.1	0.02	202.31	210	−7.69	6	6	0
3506.577	7400.338	8.36	8.4	−0.04	228.53	230	−1.47	5	5	0
3508.32	7400.315	8.39	8.4	−0.01	207.5	210	−2.5	7	7	0

**Table 3:** Correction coefficient of massive power touch rod.

	*N′*_63.5_	10	15	20	25	30	35	40	≥50
*l*									
≤ 2		1	1	1	1	1	1	1	1
4		0.95	0.93	0.92	0.9	0.89	0.87	0.86	0.84
6		0.90	0.88	0.85	0.83	0.80	0.79	0.78	0.75
8		0.86	0.83	0.80	0.77	0.75	0.73	0.71	0.67
10		0.83	0.79	0.75	0.72	0.69	0.67	0.64	0.61
12		0.79	0.75	0.7	0.67	0.64	0.61	0.59	0.55
14		0.76	0.71	0.66	0.62	0.58	0.56	0.53	0.50
16		0.72	0.67	0.62	0.57	0.54	0.51	0.48	0.45
18		0.70	0.63	0.57	0.53	0.49	0.46	0.43	0.40
20		0.67	0.59	0.53	0.48	0.44	0.41	0.39	0.36

**Table 4:** Data of the third inspection results.

Test Detection Depth (m)	Average Value after Correction	Average Hammer Value of 6 m Range below Consolidation Surface	Test Detection Depth (m)	Average Value after Correction	Average Hammer Value of 6 m Range below Consolidation Surface
5.6	9.2	7.5	7.7	8	7
6.2	8.2	7.5	7.4	11.6	6.5
6.1	10.9	7.1	8.1	11.9	7.9
6.1	9.8	6.8	8	12.7	7.7
6.3	10.4	6.1	4.3	11.9	6.8
6.9	10.7	6.4	7.2	6.2	7.4
6.3	10.9	7	5.6	11.9	6.2
5.4	10.2	6.4	8.2	8.9	7
5.1	9.4	6.5	5.3	13.5	6.9
4.8	8.8	7	5.6	9.2	6.6
5.3	7.4	6.5	7.5	8.9	6.6
6.1	6.3	6.5	6.1	11.5	7
6.1	9.5	6.6	5.1	10.6	6.3
5.4	9.6	7	7.9	8.8	7.2
6.1	9.5	7	6.9	12.4	6.3
6.3	10.2	8	7.4	7.8	6.6
5.9	9.5	7.2	7.2	11.8	6.7
7.1	9.2	7.3	5.6	8	6.9
5.5	10.8	6.6	7.4	8.9	7.1
8.4	8.6	7.1	8.3	11.5	7.3
8.1	12.5	7.8	6.6	13.4	7.1
7.6	10.4	7.2	7.7	11	8.1
5.6	12.3	7.7	7.7	12.1	7.8
5.6	9.3	7.6	5.3	12	7.5
8.3	6.9	7	6.3	9.7	8.4
8.5	9	6.4	6.5	11.6	7.9
8.1	10.8	7	5.6	10	7.6
8.3	11.4	7.2	6.2	9.2	7.7
8.2	12.4	7.5	5.2	9.1	7.1
6.9	12.7	8.2	6.3	8.1	6.6
7.2	9.8	7.6	7	7.8	5.8
7.1	10.6	7.2	7.1	9	6.2
7.3	9.6	7.5	5.9	10.1	6.8
8.1	11	8.3	6.1	9.4	7.9
5.4	11.9	7.2	6.1	8.9	6.2
8.6	8.4	6.5	6.8	10.2	8.1
7.6	12.5	6.5	6.5	10.7	8.4
6.9	11.7	6.6	6.5	10.4	8
7.2	11	6.4	5.1	9.6	7.3
